# Contemporary Trends in Pulsed Field Ablation for Cardiac Arrhythmias

**DOI:** 10.3390/jcdd12010010

**Published:** 2024-12-30

**Authors:** Hagai Yavin, Mark Prasad, Jonathan Gordon, Tolga Aksu, Henry D. Huang

**Affiliations:** 1 Department of Cardiology, Rush University Medical Center, Chicago, IL 60612, USA; hagai_d_yavin@rush.edu (H.Y.); mark_r_prasad@rush.edu (M.P.); jonathan_s_gordon@rush.edu (J.G.); 2 Department of Cardiology, Yeditepe University Hospital, 34755 Istanbul, Turkey; aksutolga@gmail.com

**Keywords:** pulsed field ablation, cryoablation, radiofrequency, atrial fibrillation, ventricular tachycardia

## Abstract

Pulsed field ablation (PFA) is a catheter-based procedure that utilizes short high voltage and short-duration electrical field pulses to induce tissue injury. The last decade has yielded significant scientific progress and quickened interest in PFA as an energy modality leading to the emergence of the clinical use of PFA technologies for the treatment of atrial fibrillation. It is generally agreed that more research is needed to improve our biophysical understanding of PFA for clinical cardiac applications as well as its potential as a potential alternative energy source to thermal ablation modalities for the treatment of other arrhythmias. In this review, we discuss the available preclinical and clinical evidence for PFA for atrial fibrillation, developments for ventricular arrhythmia (VA) ablation, and future perspectives.

## 1. Introduction

Pulsed field ablation (PFA) is a catheter-based procedure that utilizes short high voltage and short-duration electrical field pulses to induce tissue injury. Recent renewed interest has led to the emergence of the clinical use of PFA technologies and research into broadening clinical applications for PFA as an alternative to thermal ablation modalities. Although early clinical studies appear to show that PFA is effective and efficient for catheter-based pulmonary vein isolation (PVI) for atrial fibrillation (AF), there remains some uncertainty in terms of long-term clinical effectiveness, safety, and incremental benefit over current ablative technologies. In this review, we discuss the available preclinical and clinical evidence for PFA for atrial fibrillation, developments for ventricular arrhythmia (VA) ablation, and future perspectives.

### 1.1. The Early Era of Catheter Ablation for Treatment of Arrhythmias

Nonsurgical percutaneous catheter ablation for the management of cardiac arrhythmias was first reported in 1982 in a case series documenting the use of direct current (DC) shock ablation in the His bundle region for the treatment of refractory supraventricular tachyarrhythmias [1]. These procedures were performed using transvenous electrode catheters to deliver high-energy DC shocks and create ablation lesions via direct electrical injury to the cellular membrane at the intracardiac targets of interest [2,3]. The development of this technique was revolutionary in the management of tachyarrhythmias as prior treatment primarily consisted of medical therapy with limited options, or surgical ablation or substrate resection for refractory cases [4]. However, DC ablation was associated with safety concerns due to the associated barotrauma, fulguration, and proarrhythmic effects [5,6].

By the early 1990s, radiofrequency (RF) energy had become the predominant percutaneous ablation modality [7,8]. RF ablation (RFA) produces its effect through the delivery of electrical current at the catheter electrode-tissue interface leading to hyperthermic cellular injury with less skeletal muscle stimulation and need for general anesthesia, and more favorable efficacy and safety [3,9]. High temperatures produced at the catheter tip during RFA were initial limitations; however, the development of cooling techniques with irrigated ablation catheters allowed for higher energy delivery and the creation of larger and deeper ablation lesions while lowering the concomitant risk of complications from tissue hyperthermia and char formation [10,11]. Cryoablation has also emerged as one of the dominant percutaneous ablation techniques, particularly for PVI, with many studies demonstrating its effectiveness and safety [12,13,14,15,16]. Cryoablation lesions are created through direct cellular injury via the generation of extracellular and intracellular ice and microvasculature injury and are associated with a lower risk of thrombus formation when compared to RFA [17,18,19,20,21]. Recently, a new cryoballoon ablation system (PolarX, Boston Scientific, MA, USA) was introduced into commercial use with equivalent clinical effectiveness [22,23,24]. A novel ultra-cold cryoablation has been investigated, demonstrating its feasibility and safety for the treatment of atrial fibrillation (AF) and ventricular arrhythmias but is not yet commercially available [25,26]. To date, RFA and cryoablation have remained the predominant catheter ablation modalities in clinical use worldwide with multiple studies comparing their efficacies for various arrhythmias [27,28,29,30].

Additional sources of energy that have been examined for catheter ablation use include microwave, ultrasound, and laser. Microwave energy produces thermal ablation lesions through frictional and radiant heating, differing from resistive heating induced by RF energy [31,32,33]. In vitro data suggest that microwave ablation may be effective in endocardial pulmonary vein isolation (PVI) [34,35]. High-intensity focused ultrasound (HIFU) is another thermal ablation modality which achieves comparatively more lesion depth than other available energy sources [36]. Unfortunately, HIFU ablation for PVI was associated with increased rates of phrenic nerve palsy and atrioesophageal fistulas, and as a result it is no longer used for this indication [37,38]. In vivo data in swine show that HIFU may be a useful tool for the ablation of ventricular arrhythmias with mid-myocardial or epicardial sites of origin, though further study is needed [39]. Laser ablation has also been investigated as a thermal ablation energy source, with early studies comparing its efficacy for PVI to RFA and cryoablation [40,41,42,43]. The commercially available laser balloon system (Cardiofocus, Marlborough, MA, USA) for PVI utilizes a D_2_O clear liquid-filled, compliant adjustable-sized balloon to fit various PV shapes and sizes. Laser energy is delivered under endoscopic visualization by an optical laser generator focusing peak energy a few mm (infrared frequency range) subendocardially with lesions generated by radiant heating, lowering the risk of steam pops and thermal latency once energy delivery is stopped and lowering the risk of collateral injury to adjacent structures [44]. A recent study examining acute and long-term results demonstrated that the reconnection rate was low, and AF recurrence was favorable on long-term 5-year follow-up [45]. The current generation laser balloon catheter utilizes a compliant adjustable-sized balloon with endoscopic visualization during ablation and features motorized steering for continuous 360-degree laser applications that have improved first-pass isolation (FPI) and procedural times [46,47].

**Figure 1:**
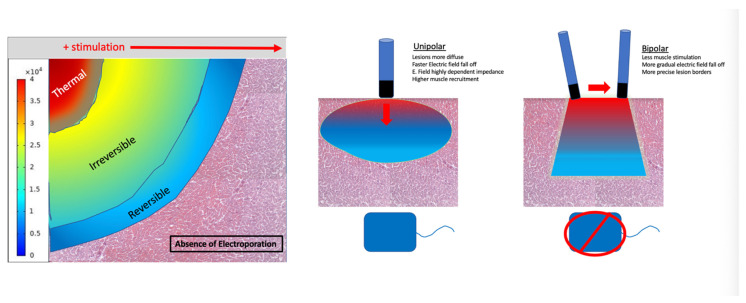
Conceptual figures showing an association between proximity of the electrode and relationship between strength of the electric field which may result in irreversible and reversible cellular electroporation. Theoretical differences between unipolar and bipolar configurations on biophysics of pulsed electric field delivery.

As the incidence of AF and the number of AF ablations performed worldwide has grown over the last few decades [48], the need for increased procedural efficiency has driven the development of new workflows and technologies [49,50,51,52,53,54,55,56,57,58,59,60,61,62,63]. Despite these advances, the risk of rare but serious complications such as phrenic nerve injury and esophageal fistula remain [64,65,66,67]. Thus, the usage of short-duration electrical energy applications has been reassessed as an ablative modality in the form of PFA. PFA uses pulsed electrical fields (PEF) to produce cell membrane injury via the creation of nanopores (irreversible electroporation) with initial clinical trials showing promising results (Figure 1) [68].

### 1.2. Pilot Pulsed Field Ablation Studies

Three of the first clinical trials that examined the feasibility of PFA for in-human use were the IMPULSE, PEFCAT, and PEFCAT II trials [69]. These prospective and multicenter studies included 121 patients with drug-resistant paroxysmal AF who underwent PVI via PFA. Invasive remapping was performed in 110 patients (90.9%) 2–3 months following ablation. Durable PVI was observed in 64.5% of patients at time remapping with 78.5% freedom from atrial arrhythmia at 1 year. Although, it cannot be excluded that the clinical performance in these trials was influenced by redo PVI within 2–3 months which does not routinely happen in clinical practice; a subgroup analysis of patients who underwent single ablation procedures showed a similar rate of freedom from any atrial arrhythmia (79.3%). Long-term follow-up data were available for 116 (95.9%) patients and showed that 73.3% remained free from any atrial arrhythmias over 49 (IQR: 45–54) months [70]. Procedure-related complications in these trials were 2.5% (Table S1).

The PULSED-AF trial was another in-human clinical trial published in 2023 that evaluated the efficacy and safety of PFA-PVI [71]. The prospective multicenter study included 300 patients with paroxysmal and persistent AF. This trial demonstrated efficacy rates for successful PVI (100%) and freedom from recurrent atrial arrhythmias at one-year follow-up of 69.5% in the paroxysmal AF cohort and 62.3% in the persistent AF cohort [13,72]. Ablation time was relatively short in both the paroxysmal (58 ± 28 min) and persistent AF cohorts (64 ± 28 min), and overall adverse events were just 0.7% in the trial (Table S1).

The MANIFEST-PF study was a multicenter retrospective survey of 1758 patients which examined the procedure characteristics and outcomes of AF ablation via PFA [73]. Patients with paroxysmal, persistent, and longstanding persistent AF were included, with PFA being the first ablation procedure for 93.5% of the cohort. The mean procedure time was 65 min with a 99.9% acute PVI success rate. The most common adverse events were vascular access complications (3.18%), pericardial tamponade (0.97%), and stroke/TIA (0.51%). Death occurred in one patient (0.06%) secondary to post-procedure stroke. There were eight occurrences (0.46%) of transient phrenic nerve injury but none that persisted beyond hospital discharge. There were no occurrences of atrioesophageal fistulas or pulmonary vein stenosis.

The above studies show that PFA is a promising modality for PVI in terms of both efficacy and safety. Large-scale clinical trials investigating the feasibility of PFA for use in non-PVI lesion sets and non-AF arrhythmias are also needed before its use can be expanded.

### 1.3. Clinical Data for Treatment of Atrial Fibrillation—1st Generation PFA Catheters

#### 1.3.1. PulseSelect PFA System

The PulseSelect PFA System (Medtronic, Minneapolis, MN, USA) consists of nine fixed electrodes spaced along a 25 mm diameter loop. After initial safety data from the PULSED AF Pilot trial, the outcomes of the system were reported in the prospective, multicenter, single-arm PULSED AF Pivotal Trial [74]. The primary endpoint, freedom from the composite of acute procedural failure, arrhythmia recurrence, or antiarrhythmic escalation at one year after a 3-month blanking period was achieved in 66.2% of participants with paroxysmal AF, and 55.1% of participants with persistent AF. Freedom from any atrial arrhythmia was also higher in patients with paroxysmal compared to persistent AF (69.5% vs. 62.3%, respectively). There was a low reported rate of adverse events overall (0.7%), with one CVA and one pericardial effusion requiring drainage.

#### 1.3.2. FARAPULSE PFA System

The FARAPULSE PFA system (Boston Scientific, Marlborough, MA, USA) utilizes a penta-spline catheter in which each of the five spines can be variably deployed to adapt to the left atrial anatomy. The system’s safety and efficacy were first shown in the IMPULSE, PEFCAT, and PEFCAT II trials [29], but the system did not receive FDA approval until January 2024 based on the results of the ADVENT trial which was a randomized, single-blinded, non-inferiority trial comparing the use of the FARAPULSE system (*n* = 305) to thermal ablation (RFA or cryoablation; *n* = 302) in patients with drug refractory paroxysmal AF [75,76]. The primary efficacy endpoint was achieved in 73.3% who underwent PFA and 71.3% who underwent thermal ablation. The study also met its primary safety endpoint, with events occurring in 2.1% of the PFA arm and 1.5% of the thermal ablation arm. The total procedure time was lower with PFA (105.8 min) compared to thermal ablation (123.1 min). However, PFA did require longer fluoroscopy time than thermal ablation (21.1 vs. 13.9 min, respectively). The acute outcomes from FARADISE, a prospective, post-market registry of the FARAPULSE PFA system, were recently presented as a late breaking abstract [77]. In the 986 participants enrolled to date, 32 (3.1%) had device- or procedure-related adverse events, including one hemolysis, one air embolism, one stroke, two pericarditis, one pericardial effusion, and one tamponade. In another smaller trial, the FARAPULSE system showed safety and efficacy in those with persistent atrial fibrillation undergoing PVI and posterior wall ablation [78].

Outcomes from the FARAPULSE system have now been reported in post-market registries, allowing for the further evaluation of outcomes and the detection of rare adverse events. MANIFEST-PF found higher freedom from atrial arrhythmia in all-comers with paroxysmal AF compared to those with persistent AF (81.6% vs. 71.5%; *p* = 0.001). However, there was no difference in freedom from atrial arrhythmias in those not taking AADs (71.3% vs. 73.5%, *p* = 0.15) [79]. In a meta-analysis of six studies comparing 1012 patients who underwent AF ablation with PFA (*n* = 441) and thermal energy sources (*n* = 571), no significant difference in the recurrence of atrial tachyarrhythmias was observed (RR 0.64, 95% CI 0.31, 1.34); however, procedure time was shorter in the PFA arm, while fluoroscopy time was also longer than for the thermal ablation arm [80]. There was also no difference in periprocedural complications (RR 1.2, 95% CI 0.59–2.44); although recently, a unique concern has arisen with reports that hemolysis is a risk of repetitive PFA applications. The degree of hemolysis in PFA was described by Osmancik et al. in their single-center prospective trial, including 70 participants undergoing first-time ablation for AF. Red blood cell microparticles increased almost 12-fold in the PFA group compared to only about 2-fold in the RFA group, although both groups returned to normal levels post-procedurally. PFA also had a higher concentration of LDH and indirect bilirubin with lower haptoglobin compared to RFA [81]. While no participants developed clinically significant renal injury in the study, cases have been reported in other post-approval registries. In the MANIFEST 17K registry, a retrospective registry of more the 17,000 patients who underwent PFA using the FARAPULSE system, 0.03% (5/17, 642) patients had hemolysis-related acute renal failure requiring dialysis [82]. Oliguria or anuria was noted post-procedurally or the following day in all cases. The mechanism is thought to be related to the electroporation of erythrocytes. Of note, the rate of renal injury not requiring dialysis has not been reported in the literature. Again, no cases of esophageal complications, pulmonary veins stenosis, or persistent phrenic nerve palsy were documented in this large post-market registry [82].

An important remaining question is the durability of PV isolation following PFA procedures. In the last few years, multiple observational European studies have now reported data on the rates of chronic PV isolation in the clinical setting during redo procedures with invasive remapping [83,84,85,86,87,88,89]. Of the 1615 patients who under PVI using PFA, 111 (6.9%) patients underwent redo ablation procedures. On remapping, 64 of 111 (58%) patients had at least one PV reconnection following ablation with the Farapulse PFA system comparable to thermal energy modalities.

### 1.4. New Pulse Field Catheters for Atrial Fibrillation Ablation

#### 1.4.1. VARIPULSE PFA Catheter

The ADMIRE Pivotal Trial presents the initial outcomes from the use of the novel VARIPULSE variable-loop PFA catheter (Biosense Webster, Irvine, CA, USA) with an integrated mapping system (Reddy September 2024). In this prospective, multicenter trial, 277 participants underwent PFA. The system showed similar rates of freedom from atrial arrhythmias at 1 year (75.4%) as other PFA catheters. However, 25% of cases were performed without fluoroscopy. The median procedure time with the VARIPULSE system was 81 min (interquartile range of 61–112 min with an adverse event rate of 2.9% [90].

#### 1.4.2. Sphere-9 Catheter

The Sphere-9 is a 7.5 F bidirectional RFA/PFA catheter with a compressible 9 mm nitinol lattice tip with nine nodes containing mini-electrodes and thermocouples (Affera, Medtronic, Minneapolis, MN, USA). In a prospective, multicenter single-arm clinical study, 178 patients with paroxysmal and persistent AF underwent PVI and linear lesion sets in a combination of lesions using RF or PFA energy at the operator’s discretion. Overall, 122 of 178 patients underwent protocol-mandated remapping procedures at a mean of 96 days post-procedure. Across the cohort, 58% of patients had all PVs still isolated; however, chronic isolation was 96–97% for patients who received the optimized PULSE3 waveform according to the investigators. No patients had PV stenosis or phrenic nerve injury, although 6.7% of patients had silent cerebral lesions on brain MRIs [91]. The SPHERE Per-AF Pivotal Trial compared the Sphere-9 Catheter and Affera mapping system to the conventional Thermocool SmartTouch SF with a Carto 3 mapping system in 432 patients with persistent AF. At 1-year follow-up, freedom from atrial arrhythmias was non-inferior (76.7%) compared to the control arm (72.8%). The total energy application time and skin-to-skin procedural time was lower in the investigational arm. In this study, 26 patients underwent redo ablation procedures (Table S1). Chronic durability was 50% in the investigational arm compared to 18.8% in the control arm [92].

A recent in vivo study examined a novel over-the-wire 8F catheter meant for single-shot PVI (Sphere-360, Medtronic, Minneapolis, MN, USA), which has a compressible lattice tip with six nodes for PEF delivery that is also expandable up to 34 mm [93]. The single-shot designed expandable lattice-shaped catheter was able to obtain the successful acute isolation of the SVC and RSPV in just 2.8 ± 1.1 and 3.2 ± 1.2 applications, respectively, in 12 swine. All targeted veins remained isolated at a mean of 23 days. No phrenic nerve injury was observed in this study, although one animal developed persistent sinus node arrest requiring pacemaker placement.

#### 1.4.3. Volt PFA Catheter System

The Volt PFA system (Abbott, Minneapolis, MN, USA) is an investigation of the 12.5 F over-the-wire balloon-in basket-PFA catheter, which is directed using a 13 F steerable sheath and is compatible with the Ensite XP 3D mapping system (Abbott, Minneapolis, MN, USA). The balloon is expandable through the injection of saline or saline/contrast mixture, and PFA energy is delivered as a bipolar biphasic PEF from 8 equally spaced splines made of insulated nitinol. Waveform power may be toggled as the low-voltage waveform is recommended in areas where phrenic nerve capture cannot be avoided. The VOLT CE Mark study was a pre-market prospective, single-arm multicenter study (*n* = 32) examining the safety and clinical effectiveness of the Volt ablation system in patients with paroxysmal and persistent AF [94]. Mean procedure time was 124.6 ± 28.1 min. On average, 23.8 ± 4.2 PFA applications were delivered, and the LA dwell time was 53.0 ± 21.0 min. No patients experienced major procedure-related complications (Table S1).

#### 1.4.4. X4 PFA Balloon Catheter System

**Figure 2:**
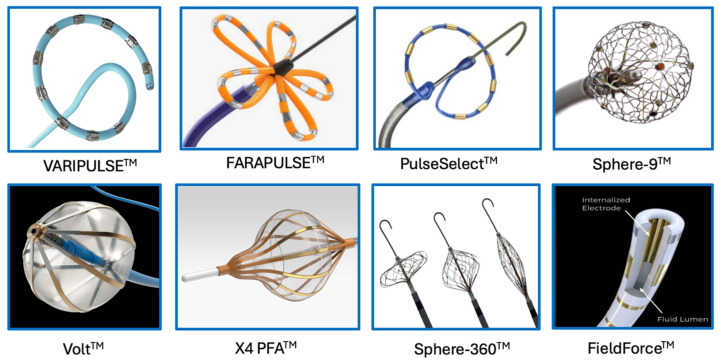
Clinical and investigation catheter technologies for pulsed field ablation.

The X4 PFA catheter system comprises a soft-tipped compliant adjustable-sized balloon catheter with endoscopic visualization via a 2F inner endoscope and the Heartlight console (Carrdiofocus, Marlborough, MA, USA). The catheter has 12 outer splines with electrodes, which are capable of delivering biphasic bipolar PEFs via the Centauri CF PFA generator (Cardiofocus, Marlborough, MA, USA). Unique features of the catheter system are the compliant balloon which may expand to up to 42 mm in diameter, and endoscopic visualization ensures the circumferential contact of the walls of the balloon (hence, electrodes) on the endocardial surface, maximizing the penetration of the electric fields during PFA delivery while also minimizing hemolysis with the visually confirmed displacement of blood. Thisl device is currently undergoing pre-clinical testing as a single-shot device that does not require repositioning or rotation between PFA applications as opposed to other devices. First-in-human studies are underway with the pivotal study expected to enroll in 2025 (Figure 2).

### 1.5. Insights from Preclinical Data on Pulsed Field Ablation for Cardiac Ablation

Over the past two decades, various studies have utilized several animal models, predominantly porcine and canine, to evaluate and better understand the biophysics and the safety and efficacy of PFA. These studies rendered promising findings that allowed further investigational work to be conducted in clinical settings.

Irreversible electroporation (IRE) as a cardiac ablation modality was first reported as early as 2007 by Lavee et al. [95], where the investigators applied IRE ablation to the left and right atrial epicardial tissue in five pigs, using a handheld clamp containing two parallel electrodes. During PFA application, no temperature change was noted in any of the animals. Histopathological assessment showed that IRE resulted in a sharp demarcation line between the lesions and the surrounding normal myocardium. These histological findings were consistent throughout various publications, all showing preferential cardiomyocyte death with a preserved extracellular structure and the sparing of noncardiac intralesional cells such as blood vessels and nerves embedded within the affected lesion area [96,97]. The feasibility of PFA application for PVI ablation was demonstrated by Witkumpff et al. in 2011 using a pig model and an endocardial circular PFA catheter. After a survival time of 3 weeks, histological findings demonstrated durable lesions to the PV ostia with a lesion depth of up to 3.5 mm.

The same group have also addressed the concerns revolving PVI stenosis, a major complication associated with RF ablation, using a swine model PV angiogram before, immediately after, and post-3-month survival period. This study showed a decrease in PV diameter with both PFA and RF immediately after ablation; however, after 3 months, the PV diameter increased in PFA lesions compared to continued stenosis seen in RF PVs [98]. This finding has been validated later in several studies with various catheters developed for single-shot PVI applications [93,99,100,101].

**Figure 3:**
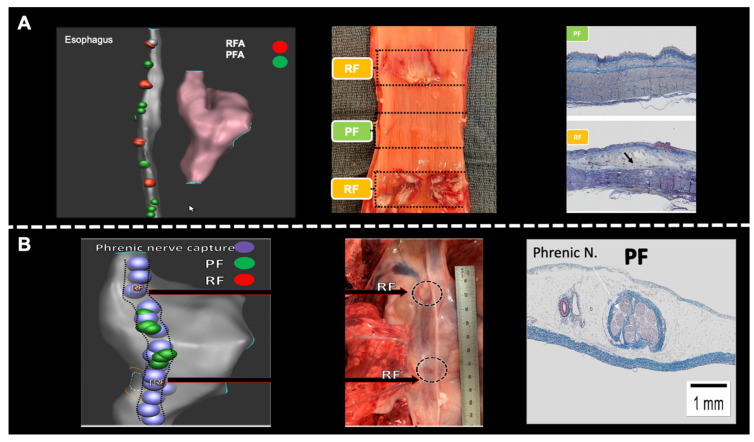
(**A**) In four swine, direct ablation with PFA and RFA within the lumen of the esophagus were performed to assess the effect of PFA on esophagus tissue. **Left**, 3D anatomical map of the esophagus and RA. Red dots represent RFA while green dots, PFA. **Middle,** gross pathology demonstrates direct ablation to the esophageal lumen, interchangeably with PFA and RFA. **Right**, histological slides of PF and RF lesions show mild edema and focal superficial necrosis in PFA lesions, while RFA shows severe edema, necrosis, and hemorrhage spanning to the deep muscularis layers. (**B**) In six swine, 5.5 (1–8) PFA applications were placed on the endocardial RA, opposing the phrenic nerve. These did not result in phrenic nerve paralysis. Comparison RF ablation. **Left**, anatomical map with the course of the right phrenic nerve identified by pacing the lateral RA marked in light-blue tags. Green tags represent PFA and red represent RFA. **Middle**, gross pathology of the phrenic nerve with clear lesions at RFA sites opposed to the healthy-looking tissue at the PFA sites. **Right**, histological analysis at PFA application sites demonstrating PFA selectively affected cardiomyocytes but spared blood vessels and nervous tissue.

The safety of PFA was also established with regard to the phrenic nerve and esophagus, both organs known to be susceptible to RFA injury and serious complications such as atrio-esophageal fistula and phrenic nerve palsy. Several animal models have been applied to assess esophageal injury. Koruth et al. [102]. have used an esophageal deviation ballon to deflect the lower esophagus toward the inferior vena cava (IVC). In 10 swine, RF and PF ablation was conducted at distinct positions within the IVC that were in contact with the esophagus. Esophageal injury was seen in all RFA animals, while none of the PFA animals demonstrated esophageal lesions at 25 days. In one out of the four RF animals, esophago-pulmonary fistula and a deep esophageal ulcer were found. In another study using direct intraluminal ablation in the esophagus with a 24 h survival period, PFA resulted in mild edema and the focal minimal necrosis of superficial epithelium, while RFA resulted in severe edema, inflammation, necrosis, and hemorrhage spanning to the deep layers of the esophagus [103] (Figure 3A).

Van driel et al. [104] demonstrated continued phrenic capture in 17 out of 19 animals immediately after PFA applications at a level above the lesion site and in all animals after 30 min. Another study delineated the course of the phrenic nerve in 10 swine. In six animals, a normal clinical PFA dose was directly applied to the phrenic nerve, while in the other four, a supratherapeutic dose was applied. In the normal dose group, no phrenic nerve stunning was seen; however, in the supratherapeutic dose, one out of the four animals developed phrenic nerve stunning that resolved within 5 min. At remapping, all animals had intact phrenic nerve capture at 25 days. Histology analysis demonstrated normal nerve bundle appearance in PFA sites [105] (Figure 3B).

With the increasing pre-clinical and promising clinical results of both the efficacy and safety of PFA on atrial tissue, increased interest promoted studies looking into the effect of PFA in achieving larger and deeper lesions in ventricular tissue. Early studies from as soon as 2012 focused on epicardial surgical lesion applications [106]. Using a chronic 3-week survival swine model, histological analysis demonstrated that lesion depth is directly proportional to the strength of the current used for PFA. Since most early publications utilized the epicardial approach, a major area of interest was the effect of PFA on the coronary arteries. Early publications demonstrated a promising absence of short- and long-term luminal narrowing after PFA applications [107,108]. Neven et al. described the long-term effect of epicardial IRE application on coronary artery luminal diameter. In six pigs, surgical epicardial applications were performed on the LV epicardium in the vicinity of the coronary arteries. An angiogram was conducted before, immediately after, and 3 months post-ablation. IRE applications led to short-lasting coronary spasms with no long-term luminal narrowing of the coronary arteries at the areas of clear ablated myocardial tissue.

Endocardial approaches were also investigated in pre-clinical studies in recent years. Yavin et al. [109] have demonstrated in a porcine model that lesion repetition may lead to a substantial increase in lesion dimensions in ventricular tissue. Recent publications have focused on animal infarcted/scarred myocardia. Gerstenfeld et al. [110] have utilized an anterior LAD infarct model in 10 animals with a post-infarct survival of 6–8 weeks.

After survival, PFA and RFA applied onto scarred tissue revealed a greater lesion depth while utilizing PFA (PFA vs. RFA; 6.1 ± 1.7 mm vs. 3.8 ± 1.7 mm; *p =* 0.005). Younis et al. [111] confirmed these results in a similar model and elucidated that PFA resulted in well-demarcated and uniform lesions exhibiting cardiomyocyte death, contraction bands, and lymphocytic infiltration, which are characterizations of IRE. The effect of PFA extended through collagen and fat to the epicardial layers, in contrast to the RFA that showed less uniform and limited effect to the subendocardial layers with a sparing of deeper viable myocardium within scarred tissue.

### 1.6. Cardiac Neuromodulation Effect of Pulsed Field Ablation

Previous experimental studies determined that clusters of autonomic epicardial ganglia derived from the intrinsic cardiac autonomic system (ICANS), known as ganglionated plexi (GPs), were critically involved in the initiation and maintenance of AF [112,113]. It has been demonstrated that PVI with thermal energy sources results in a partial GP ablation or neuromodulation which may contribute to PVI success [114,115,116]. Recently, published studies have demonstrated that GP ablation in addition to PVI may increase AF-free survival compared with PVI alone [114,117,118,119]. As a nonthermal modality, while PFA causes irreversible damage to the myocardium using direct current electrical pulses, nerves and other collateral structures are spared due to high tissue selectivity. Thus, the following questions need to be clarified: (1) what is the acute and long-term effect of PFA on the GP and the ICANS? (2) Will neuromodulation capability of PFA affect success of PVI in AF?

In 2022, Stojadinović et al. [120] compared the alteration of the ICANS after PVI using PFA and RFA. The response of the sinoatrial node (SAN) and atrioventricular node (AVN) to extracardiac vagal stimulation (ECVS) was evaluated at baseline, during, and at the end of the ablation procedure. As an acute effect, RFA caused a higher reduced response in both SAN and AVN (100% vs. 33%, *p* = 0.0001 in SAN, and 93% vs. 33%, *p* = 0.002 in AVN, respectively). The early recovery of the GP function was noticed only in the PFA group. RFA resulted in the higher acceleration of the sinus rhythm compared with PFA (20 ± 13 beats/min vs. 12 ± 10 beats/min, *p* = 0.04). Del Monte et al. [121] investigated the degree and acute vagal modulation induced by the PFA during PVI compared with cryoballoon ablation (CBA). The effect on the ICANS in the two groups was assessed at three pre-defined timepoints with ECVS: (1) before PVI; (2) immediately after PVI; and (3) 10 min after the last energy application. The vagal response induced by ECVS after PVI almost disappeared in the CBA group but persisted in the PFA group. Intraprocedural vagal responses occurred more frequently with PFA than CBA. Moreover, HR 24 h post-PVI increased more with CBA than with PFA. ECVS demonstrated that PFA determined a significant acute suppression of the vagal response immediately after PVI.

Guo et al. [122] evaluated the autonomic nervous system effect of a PFA system using nerve injury biomarkers (serum neurofilament light, glial fibrillary acidic protein, S100β, and brain fatty acid-binding protein) and heart rate variability (HRV) during PVI. There were no significant changes in serum nerve injury biomarkers in pre-ablation and immediately post-ablation, and 24 h after ablation. Similarly, there was no significant change in pre-ablation and 30-day post-ablation HRV parameters. Tohoku et al. [123] compared the clinical impact of PFA on ICANS with CBA by analyzing pre- and post-procedural serum S100 levels. Overall, S100 increased in both groups with a lower amount in PFA (0.035 μg/L; IQR: 0.02–0.063 μg/L) compared with CBA (0.12 μg/L; IQR: 0.09–0.17 μg/L; *p* < 0.0001). Furthermore, during PFA PVI, 30 patients (56%) demonstrated transient bradycardia in 70 of 210 pulmonary veins (35%). ΔS100 was similar between patients with or without transient bradycardia.

In a two-staged study, the impact of PFA on the GP was assessed in patients undergoing PVI [124]. In the retrospective phase, pre- versus post-PVI heart rates (HRs) were compared using PFA, CBA, or RFA. In the prospective phase, high-frequency stimulation (HFS) was used to map GPs, and vagal responses were evaluated after each set of pulsed field applications during PVI. At the end of the procedure, repeat HFS at each GP site was repeated to assess for the persistence of vagal effects. Between baseline and 3 months, increase in HR was higher after thermal ablation than PFA (8.9 ± 11.4 beats/min with RFA, 11.1 ± 9.4 beats/min with CBA, and −0.1 ± 9.2 beats/min with PFA, respectively, *p* = 0.01). In the prospective phase, pre-PFA HFS in 20 additional patients identified 65 GP sites. Although PFA caused vagal effects in 45% of first pulsed field applications which persisted through all applications in 83%, HFS post-PFA reproduced vagal effects in 29 of 38 sites (76%).

In the recently published randomized pivotal ADVENT study, PFA was compared with thermal ablation in patients with paroxysmal AF [125]. Baseline HR was derived from a pre-ablation 12-lead ECG, while follow-up HRs, as well as HRV metrics, were acquired from 72 h Holter monitors at 6 and 12 months. Thermal ablation caused a significantly greater increases in HR from baseline to 6 months and 12 months compared to PFA (∆HR; 10.1 vs. 5.9 beats/min; *p* = 0.02, and ∆HR; 8.8 vs. 5.2 beats/min; *p* = 0.03, respectively). HR effects were similar between CBA and RFA at 6 and 12 months (*p* = 0.94 and 0.83, respectively). HRV metrics were significantly lower at both 6 and 12 months after thermal ablation compared to PFA (*p* < 0.01). In a similar way, Valeriano et al. [126] assessed the effects of PFA and RFA on GPs ablation by examining HR and HRV dynamics in a total of 105 patients undergoing PVI. ECGs were collected before and after ablation at multiple timepoints—1 h, 1 day, 1 month, and 3 months—with a 24 h Holter ECG at 3 months. While HR significantly increased at all measured timepoints compared with baseline in the RFA group, PFA had no effect on HR, and this difference persisted after 3 months. The RFA group showed significantly lower HRV indices 3 months after PVI.

**Figure 4:**
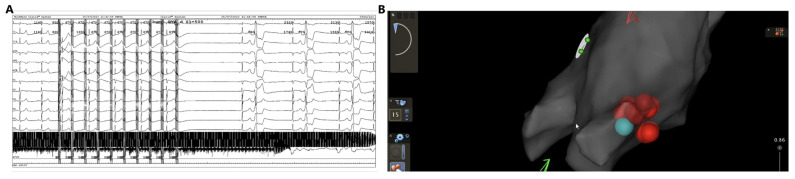
(**A**) First pulsed field ablation (PFA) application from left atrium near right superior pulmonary vein induces a profound vagal response. (**B**) The red spherical tags show radiofrequency lesions given nearby the PFA lesion where acute vagal response was obtained which are indicated with blue and green spherical tags (from superior view). Although radiofrequency (RF) lesions did not induce further vagal response after PFA application, RF applications were performed to ensure long-term parasympathetic denervation. Image reproduced with permission from Sikiric et al. *J. Interv. Card Electrophysiol.* (2024).

All these studies suggest that PFA causes inconsistent and variable degrees of acute autonomic effect on GPs, similar to high-frequency stimulation. However, a decrease in vagal effects after PFA shows a pattern of gradual attenuation, followed by a quick post-ablation recovery, whereas the vagal attenuation effect resulting from thermal ablation exhibits long-term persistence, which suggests sustained damage to the nerves with thermal ablation (Figure 4). PFA demonstrates tissue selectivity based on pulsed electric field energy settings. It is possible that different energy settings may cause a more or less neuromodulation effect which may lead to adverse ablation outcomes, as the incomplete destruction of GP can promote nerve regeneration by releasing neurotrophic factors. Hyperinnervation may create patches of heterogeneity in refractoriness within the atria and be proarrhythmogenic [127].

Utilizing the tissue selectivity of pulsed field energy may present a unique opportunity for ultraselective GP ablation without damaging adjacent pulmonary vein myocardium, and vice versa, to understand the role of neuromodulation in AF ablation outcomes. In the NEURAL AF trial, the feasibility of the epicardial ablation of the GPs with pulsed field energy during cardiac surgery was studied [128]. Patients with or without a history of AF underwent epicardial GP ablation with PFA during coronary artery bypass grafting. The atrial effective refractory period was determined immediately pre- and post-GP ablation to assess ICANS function. GP ablation resulted in a 20.7 ± 19.9% extension in the atrial effective refractory period.

**Figure 5:**
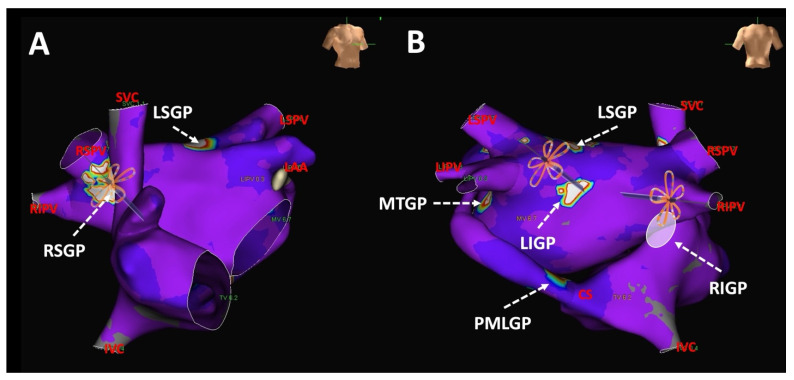
(**A**) shows penta-spline pulsed field ablation (PFA) catheter positioned at the right superior ganglionic plexus. (**B**) shows penta-spline PFA catheter position at the left superior and right inferior ganglionic plexuses.

These novel findings highlight the importance of better optimizing pulsed electric field energy for more targeted effects (Figure 5). Large, randomized controlled trials are necessary to determine the role of neuromodulation in AF ablation results, and whether different pulsed field anergy settings and application routes can offer a meaningful impact on ICANS and reduce AF.

### 1.7. Pulsed Field Ablation for Treatment of Ventricular Arrhythmias

The management of ventricular arrhythmias (VA) in patients with and without structural heart disease remains a challenge [4,129,130,131,132]. Despite technological advancements and the improved ability to better localize abnormal substrates, putative circuits, and sites where VAs originate, durable success remains elusive when the critical substrate is deep or nearby critical structures at risk of collateral damage. Although substrate modification has been increasingly adopted as an ablation strategy, the necessity for a substantial number of RF ablation lesions, often of long duration, particularly in patients with a large scar burden, often lengthens procedural and anesthesia time, leading to increased morbidity and possibly short-term mortality [133].

The biophysical limitations of therapeutic RF energy use in the ventricle are well understood. During RF delivery, a greater proportion of lesion size growth is attributed to conductive heating than resistive heating over time. The zone of lethal isotherm reaches a plateau relatively quickly due to thermal equilibrium with adjacent tissue and nearby vasculature. In order to increase lesion size, current delivery at the catheter electrode-tissue interface must be increased or RF time must be extended; however, an increase in lesion size eventually becomes incremental and the risk of tissue hyperthermia such as coagulum formation and steam pop increases further [134]. Scar tissue greatly affects thermal energy distribution leading to an unpredictable depth of penetration [111,135]. Thus, investigators have looked at alternative methods of RF delivery, although they remain bail-outs due to the risk of complications [136,137,138,139,140,141,142,143,144,145,146]. Finally, due to biophysical constraints, RF delivery is poor in certain locations such as the coronary cusps, coronary venous system, and epicardial surface where fat pads are located.

Early pre-clinical studies demonstrated the feasibility of PFA in the left ventricle (LV) [105,108,147,148]. While the development of PFA catheters for left atrial ablation have focused on minimizing injury to adjacent structures more so than necessarily maximizing lesion depth, for VAs associated with intramural substrate, achieving adequate lesion depth may be more paramount than seeking to avoid injury to non-cardiac structures, though the risk of coronary spasm likely remains [149,150]. Currently, optimal dosing strategies for PFA in the ventricle remains unclear and will need to be individually studied for different devices and anatomic locations. In theory, the relatively lower impedance of scar versus healthy tissue is less likely to affect energy field distribution during PFA delivery. In vivo studies suggest that PFA may lead to larger lesions in infarcted substrate than RF [110,151], although the dosing of RF in those studies may not have exactly emulated RF dosing strategies in clinical practice where long-duration RF applications are performed to maximize lesion depth [152]. In a recent in vivo study [151], eight swine underwent coronary balloon occlusion and ablation with an irrigated CF-sensing catheter using the CENTAURI system (Cardiofocus, Marlborough, MA, USA). A total of 53 lesions were delivered, demonstrating slightly smaller but significant lesion depth on histologic examination compared to PFA in healthy myocardium depth (5.3 ± 1.9 vs. 7.2 ± 2.1 mm, *p* = 0.0002). Some thermal effect on tissue was present after PFA, but coagulative necrosis was lower for PFA than RF lesions (15 vs. 76%).

Although the presence of edema attenuates the benefits of lesion stacking with RFA, repetitive applications of PFA appear to drive greater lesion depth than single applications, possibly through permeability preconditioning, and investigators have studied these strategies to augment success in certain anatomical locations [117]. Nies et al. examined repetitive PFA applications at epicardial and papillary muscle sites, and also the bipolar ablation of the intraventricular septum (IVS) and the LV lateral wall in the chronic swine model [153]. Papillary muscle lesions (*n* = 13) achieved a depth of 5.8 ± 1.1 mm, and epicardial lesions reached a depth of 9.1 ± 1.9 mm on average. As with RF, bipolar PFA with two opposing catheter tips yielded deeper lesions than single catheter PFA [31]. Younis et al. [154] randomized 10 swine to PFA vs. RFA at IVS, papillary muscles, epicardium, and LV summit sites through the distal coronary vs. PFA lesions, which resulted in a greater depth than RF lesions at papillary muscles, LV summit, and epicardial sites. Notably, 75% of epicardial PFA and RF applications were delivered over areas of epicardial fat. While these pre-clinical results are promising, computer modeling has previously demonstrated that the thick areas of intramyocardial fat alter electric field distribution due to their lower conductivity with lower electric field strength at entry and exit points during PFA delivery [155].

The feasibility of bipolar PFA has been demonstrated in the IVS and deep intramural locations in pre-clinical and clinical studies. In a chronic canine model (*n* = 8), Van Zyl et al. used PFA to deliver 27 ablations between two solid tip catheters, achieving a lesion depth of 10.9 mm at 4 weeks, although acute complications of ventricular fibrillation (VF) (1 animal), persistent RBBB (38%), and transient complete AV block (63%) during PFA at the anteroseptum occurred [156]. Tan et al. performed PFA using nanosecond PEF through active fixation leads placed in the RV septum using single lead in bipolar configuration and bipolar ablation between two leads with minimal skeletal muscle stimulation and without AV block. Bipolar ablation between two active fixation leads led to larger lesions than a single lead in bipolar configuration [157]. It has been postulated that the Purkinje system plays a role in the triggering and maintenance of ventricular tachycardia (VT) and VF, and the role of Purkinje de-networking has been studied [158,159,160,161,162]. Preclinical evidence suggests that PFA may selectively eliminate Purkinje fibers while sparing the aspects of the conduction system covered by myelin sheaths, potentially positioning PFA as a method to de-network the Purkinje system when appropriate for the treatment of VT/VF [163,164].

Although the clinical efficacy and safety of PFA has been examined in large registries and randomized trials in patients with AF, there are still only sparse clinical data on ventricular ablation [165,166,167,168,169,170,171,172,173]. The largest cohort consisted of a study population of 44 patients with premature ventricular complexes (PVC)s and scar-mediated VT. PFA was performed using an irrigated 4 mm ablation catheter coupled with the CENTAURI generator. In total, 84% of procedures were acutely successful (81% for clinical PVC suppression/83% for VT non-inducibility) with an average of 16 ± 15 PFA applications per case. Notably, transient complete AV block occurred in 7% of patients, which the investigators surmised was due to current leak from the proximal electrode during PFA. PVC suppression remained durable in 81% of pts at 3-month follow-up, while 52% of patients did not have a recurrence of VT on short-term follow-up (116 ± 75 days) suggesting more modest results in patients with infarct-related VT in comparison to studies utilizing RF ablation [174].

Optimal dosing strategies and catheter forms for VA ablation still need to be determined. Future focal catheter designs may favor monopolar over bipolar configurations and potentially monophasic over biphasic wave forms, as the strength and distribution electric fields tend to deliver deeper ablation lesions [175,176]. Some potential disadvantages of using monophasic waveforms may be more significant skeletal muscle and diaphragmatic stimulation, which may reduce catheter stability. Reduced electrode surface area may also increase the risk of arcing, plasma gas formation, and microbubbles during RF applications, necessitating the adjustment of dosing, adequate irrigation, and enough “resting time” to decrease residual electrode charge between PFA applications, which may blunt some of the time savings of PFA relative to conventional thermal sources [176,177]. Pre-procedural imaging to evaluate substrate composition by cardiac MRI or CT to evaluate the varying degrees of fibrosis, and the presence of fat and tissue could impact the dosing and number of PF deliveries, suggesting the benefit of a tailored approach in the ventricle. PFA performed using catheters capable of ablating larger areas may be beneficial in terms of reducing procedure time in patients with large scar burden, particularly if scar homogenization is desired [178,179,180,181,182].

Several interesting technologies for ventricular ablation are under development, specifically for PFA applications in the ventricle. The Field Force ablation system (Field Medical, Cardiff, CA, USA) utilizes a CF-sensing lumened tip catheter and propriety waveform to deliver high-intensity PEF for nearly transmural lesions in pre-clinical studies. The Ventricular Catheter Ablation Study (VCAS) is currently enrolling a prospective single-arm safety and feasibility first-in-human study with an enrollment target of 60 patients. The CFC EP ablation system consists of a generator and catheters suite consisting of a 25 mm or 30 mm, 8 Fr, 16-electrode spiral PFA catheter and a 6 Fr, linear, hexapolar PFA catheter. At 28 days, the lesion depth was 8 ± 3 mm using the spiral catheter and 8 ± 1 mm using the linear catheter [183]. Another potential technology for PFA ventricular ablation is the Focused Electric Field Ablation catheter (Focused Therapeutics, Salt Lake City, UT, USA) which utilizes an irrigated, lens-shaped concave catheter tip to deliver a collimated electric field. In preclinical studies, the catheter was shown to deliver non-surface dependent heating and transmural lesions with RF energy and is currently undergoing testing for PEF delivery in benchtop and in vivo testing [184,185].

There are some potential limitations of PFA which could affect its adoption in clinical practice. The acute loss of electrograms immediately post-PFA may not necessarily correlate with irreversible tissue injury and necrosis, which could be problematic as procedural endpoints often employed for substrate ablation include the elimination of late potentials, LAVAs, and dechanneling, which is guided by the presence and amplitude of signals within low-voltage areas. The ventricular chambers are larger than the atria, and the maximal number of PFA applications which can be safely delivered must be weighed against the risk of hemolysis and acute kidney injury. Coronary spasm is also a concern, given the patient population for whom a persistent decrease in coronary perfusion could lead to rapid deterioration and reduced hemodynamic function in the laboratory.

It remains to be seen if PFA will replace RF ablation as the primary modality for VT ablation; however, it does appear that PFA may have some advantages for certain anatomical locations or regions where higher CF cannot be achieved for RF ablation. Technologies will likely be developed in the future which will need to be assessed and tested in the clinical setting.

### 1.8. Will PFA Replace Thermal Modalities for Ablation?

While the adoption of PFA as an energy source is well on its way for AF catheter ablation, more investigation is needed to understand its role in the treatment of other clinical arrhythmias, especially given that thermal modalities have a decades-long track record of versatility and efficacy. There is also hope that future technologies and our increased understanding of the biophysical characteristics of PEF applications will address the major limitations of early PFA systems with the refinement and harmonization of future technological designs.

Although obviating the need for phrenic nerve pacing and esophageal temperature monitoring should in theory simplify workflows, deep conscious or general anesthesia is still required during PFA cases due to skeletal muscle stimulation and cough reflex during PEF deliveries with 1st generation devices, whereas ablation using moderate sedation has only been shown to be achievable with RF and cryoablation energy sources [186,187,188,189]. Although first generation PFA catheters such as Farapulse (Boston Scientifc, Marlborough, MA, USA) and PulseSelect (Medtronic, Minneapolis, MN, USA) are large profiled, neither are true single-shot catheters, and multiple applications at different locations and rotations are required to improve the chances of durable isolation. Given the direct importance of electrode proximity to lesion depth, focal catheters with CF-sensing and conformable single-shot designs may allow for the fine-tuning of more efficient waveforms, which achieve adequate lesion depth but lower proclivity for muscle stimulation, allowing for lower sedation requirements.

While the performance of first generation PFA catheters resulted in time savings for PVI compared to RF ablation with focal catheters, improvements in technology including irrigated temperature-controlled catheters, new workflows with RF, versatility for additional ablation lesion sets, and related relative cost efficiency may still favor other modalities, at least until focal and single-shot PFA catheters become available. For the ablation of atrial fibrillation, the sparing of the ganglionated plexi could perhaps adversely affect the effectiveness of PVI in some individuals; thus, patient selection will be important. Another challenging scenario would be for the ablation of AVNRT, one of the most common supraventricular arrhythmias, which requires the modification of the slow AV nodal pathway regions, due to the risk of transient and perhaps permanent AV block from the ablation of the compact AV node.

The future adoption of dual energy use PFA/RFA catheters, if effectively designed, provides operators with the promise of being able to deliver versatile lesion sets while optimizing biophysical characteristics in a given anatomical area. The Affera Sphere-9 is the first catheter technology designed specifically to seamlessly toggle between RF and PFA energy, which has proven its effectiveness and safety in clinical studies. The choice of using titratable RF energy dosing when maximal lesion depth is required or opting for the safety characteristics of PFA is an advantage of having both energy modalities in a single catheter and may favor the use of dual energy options in the future.

## 2. Conclusions

Pulsed field ablation has proven itself as a formidable, efficient, and safe modality for atrial fibrillation ablation. An increased understanding of the biophysics of PFA and new technologies opens the possibilities of using PFA as an alternative to thermal energy sources for the treatment of other tachyarrhythmias in electrophysiology.

## Supplementary Materials

The following supporting information can be downloaded at: https://www.mdpi.com/article/10.3390/jcdd12010010/s1, Table S1: Summary of Clinical Outcome Data for Pulmonary Vein Isolation with Pulsed Field Ablation Catheters in Atrial Fibrillation.
