# Supplementary material for: Contemporary Trends in Pulsed Field Ablation for Cardiac Arrhythmias
Source: J Cardiovasc Dev Dis. 2024 Dec 30;12(1):10. doi: 10.3390/jcdd12010010 (PMC11766314; doi:10.3390/jcdd12010010)
Supplement: Supplementary file 1 [file jcdd-12-00010-s001.zip › jcdd-3251573-supplementary.pdf]

Table S1. Summary of clinical outcome data for pulmonary vein isolation with pulsed field ablation catheters in atrial fibrillation

Name	Study Type	Population	PVI Method	Efficacy Outcomes	Safety Outcomes
IMPULSE, PEFCAT, and PEFCAT II 1-year Outcomes (2021)	Prospective, multicenter, nonrandomized single arm trial	Paroxysmal, symptomatic AF (n=121)	PFA with FARAPULSE PFA system, 2–3 month remapping and redo PVI with RF or PFA if reconnection	PVs remained isolated in 64.5% of patients after remapping and in 84.1% of patients who received optimized biphasic PFA waveform. Freedom from any atrial arrhythmia at one year for the entire cohort was 78.5% ± 3.8 and 84.5% ± 5.4 in those that received the optimized biphasic waveform.	Events occurred in 2.5% of participants. Two had pericardial effusions or tamponade, one had hematoma, and one had TIA.
PULSED-AF Pivotal Trial (2023)	Prospective, multicenter, nonrandomized single arm trial	Paroxysmal (n=150) or persistent (n=150) symptomatic AF despite AADs	PFA with PulseSelect PFA system	Freedom from procedural failure, arrhythmia recurrence, or AAD escalation at 1 year in 66.2% (95% CI 57.9 -73.2) of those with paroxysmal and 55.1% (95% CI 46.7-62.7) of those with persistent AF.	Events occurred in 0.7% (one in each group) with one reported CVA and one pericardial effusion requiring drainage.
ADVENT (2023)	Prospective, multicenter, randomized, single-blinded, non-inferiority trial	Paroxysmal, symptomatic AF that was drug refractory	Conventional ablation (n=302) with RF or cyroballoon vs. PFA (n=305) with the FARAPULSE PFA System	Freedom from composite of procedural failure, atrial tachyarrhythmia, AAD use, cardioversion, or repeat ablation in 73.3% participants in the PFA arm vs. 71.3% in the conventional arm.	Serious adverse events occurred in 2.3% in PFA arm (cardiac tamponade [2], death [1], pulmonary edema [1], vascular access complication [1]) and 2.0% in the thermal ablation arm (TIA [1], phrenic nerve palsy [2] with cyroballoon, pulmonary edema [1], vascular access complications [2]).
MANIFEST-PF 1- year Outcomes (2023)	Retrospective, international, post-market registry of	Participants undergoing PVI with PFA between	PFA only with FARAPULSE PFA system	Freedom from atrial arrhythmia in 78.1% (95% CI 76.0-80.0) for all participants at one year.	The major complication rate was 1.9%, including 1.1% with cardiac tamponade, 0.06% with phrenic nerve

	FARAPULSE PFA system in Europe	3/2021 and 5/2022 in Europe (n=1,568)		Treatment was more effective in those with paroxysmal vs. persistent AF (81.6% vs. 71.5%; P=0.001)	injury, 0.4% with stroke, of which one participant died (0.06%), and 0.1% had vascular access complications requiring surgery. The minor complication rate was 4%, largely driven by vascular access complications in 2.6%.
MANIFEST-17K (2024)	Retrospective, international, post market registry of FARAPULSE PFA system	Paroxysmal (57.8%) and persistent (35.2%), symptomatic AF (n=17,642) from 106/116 centers using FARAPULSE PFA system	PFA only with FARAPULSE PFA system	-	Non-energy related adverse events were 0.98%, primarily pericardial tamponade and vascular access complications. Minor complication was 3.21% also primary vascular access related. Coronary spams occurred in 0.14%, hemolysis resulting in renal failure requiring temporary dialysis occurred in five patients. The overall mortality rate was 0.03%.
FARADISE (2024)	Prospective, international, post-market registry of FARAPULSE PFA system	Paroxysmal (68%) and persistent (32%), symptomatic AF (n=986)	PFA only with FARAPULSE PFA system	-	Device and procedure-related adverse events were reported in 32 participants (3.07%). This includes access site complication (1), air embolism (1), stroke (1), hemolysis (1), pericarditis (2), pericardial effusions (2), cardiac tamponade (1)
ADMIRE Pivotal Trial (2024)	Prospective, multicenter, single arm, nonrandomized trial	Paroxysmal, symptomatic AF that was drug refractory (n=277)	PFA only with VARIPULSE PFA system and integrated mapping system	Freedom from AF, atrial tachycardia, or atrial flutter was 75.4% at one year. Composite of freedom from atrial tachyarrhythmia, failure to achieve PVI, use of non-study catheter or	The safety endpoint occurred in 2.9% (8/277). Participants had cardiac tamponade (3), major vascular access complications (2), pericarditis (1), CVA (2), and TIA (1).

				repeat procedure after blanking period was 74.6%.	
SPHERE Per-AF (2024)	Prospective, multicenter, randomized, single-blinded, noninferiority trial	Persistent, symptomatic AF (n=420)	Sphere-9 PFA catheter + RF vs. RF alone	Freedom from acute procedural failure, repeat ablation at any time, arrhythmia recurrence, AAD initiation or escalation or cardioversion after a 3-month blanking period out to one year was observed in 73.8% and 65.8% of patients in the PFA+RF and RF controls, respectively (P < 0.0001 for non-inferiority)	Safety events occurred in 1.4% of the PFA+RF arm (pulmonary edema [1] due to hypertension, COPD exacerbation [1], hemoptysis [1]) and 1.0% in the RF arm (pulmonary edema [2]) (P<0.0001 for noninferiority).
VOLTE CE Mark Study (2024)	Prospective, multicenter, single arm, nonrandomized trial	Paroxysmal and persistent, symptomatic AF (n=32)	PFA only with Volt PFA system	-	No symptomatic safety events occurred peri-procedurally or by day 7. However, screening tests discovered three participants with esophageal injury thought to be from imaging probe and three participants had silent cerebral lesions.

Atrial fibrillation = AF; Antiarrhythmic drug = AAD; Cerebrovascular accident; Pulsed field ablation = PFA; Transient ischemia attack = TIA;

Pulmonary vein isolation = PVI
